# The Increased Effectiveness of HIV Preventive Intervention among Men Who Have Sex with Men and of Follow-Up Care for People Living with HIV after ‘Task-Shifting’ to Community-Based Organizations: A ‘Cash on Service Delivery’ Model in China

**DOI:** 10.1371/journal.pone.0103146

**Published:** 2014-07-22

**Authors:** Hongjing Yan, Min Zhang, Jinkou Zhao, Xiping Huan, Jianping Ding, Susu Wu, Chenchen Wang, Yuanyuan Xu, Li Liu, Fei Xu, Haitao Yang

**Affiliations:** 1 Jiangsu Provincial Center for Disease Control and Prevention, Nanjing, China; 2 Nanjing Municipal Center for Disease Control and Prevention, Nanjing, China; 3 The Global Fund to fight AIDS, Tuberculosis and Malaria, Geneva, Switzerland; 4 Jiangsu Provincial Preventive Medicine Association, Nanjing, China; University of Missouri-Kansas City, United States of America

## Abstract

**Background:**

A large number of men who have sex with men (MSM) and people living with HIV/AIDS (PLHA) are underserved despite increased service availability from government facilities while many community based organizations (CBOs) are not involved. We aimed to assess the feasibility and effectiveness of the task shifting from government facilities to CBOs in China.

**Methods:**

HIV preventive intervention for MSM and follow-up care for PLHA were shifted from government facilities to CBOs. Based on ‘cash on service delivery’ model, 10 USD per MSM tested for HIV with results notified, 82 USD per newly HIV cases diagnosed, and 50 USD per PLHA received a defined package of follow-up care services, were paid to the CBOs. Cash payments were made biannually based on the verified results in the national web-based HIV/AIDS information system.

**Findings:**

After task shifting, CBOs gradually assumed preventive intervention for MSM and follow-up care for PLHA from 2008 to 2012. HIV testing coverage among MSM increased from 4.1% in 2008 to 22.7% in 2012. The baseline median CD4 counts of newly diagnosed HIV positive MSM increased from 309 to 397 cells/µL. HIV tests among MSM by CBOs accounted for less than 1% of the total HIV tests in Nanjing but the share of HIV cases detected by CBOs was 12.4% in 2008 and 43.6% in 2012. Unit cost per HIV case detected by CBOs was 47 times lower than that by government facilities. The coverage of CD4 tests and antiretroviral therapy increased from 71.1% and 78.6% in 2008 to 86.0% and 90.1% in 2012, respectively.

**Conclusion:**

It is feasible to shift essential HIV services from government facilities to CBOs, and to verify independently service results to adopt ‘cash on service delivery’ model. Services provided by CBOs are cost-effective, as compared with that by government facilities.

## Introduction

Since the first case of HIV/AIDS was reported in China in 1985, the epidemic has spread to different populations around the country [1], [2]. HIV transmission route has also shifted from predominantly injection drug use (IDU) during the late 1980s to early 1990s, to unsafe blood and plasma collection in mid-1990s, and to the sexual contact mode since 2005 [3]. Among new annual infections estimated in 2011, 52.2% were infected through heterosexual contact and 29.4% through homosexual contact [4].

Similar to control programs for other infectious diseases, programs for HIV/AIDS shall follow public health principles, including case finding and surveillance, interrupting transmission, treatment and case management, together with population based monitoring and evaluation [5]. Successful adoption of these public health principles requires a non-stop service chain ranging from preventive intervention to avoid HIV acquisition, to HIV testing to detect the infection, prevention of secondary transmission with positive prevention, follow-up care, preparation and initiation and retention with antiretroviral therapy (ART) [6].

China’s central government and local governments have taken various measures to contain the epidemic. These include HIV testing, preventive interventions such as methadone maintenance therapy and behavioral change, prevention of HIV mother-to-child transmission, follow-up care for people living with HIV/AIDS (PLHA) and implementation of free ART [6], [7]. These government funded HIV programs are implemented by health and medical professionals within the government structure, primarily through its public health system, namely national -provincial-prefecture- county/district center for disease control and prevention (CDC) system. This arrangement, in the context of the stigma and/illegality being associated with high risk behaviors and with being HIV positive, hindered the acceptability and uptake of relevant HIV program services [8]–[11]. The difference between the two numbers of 343,000 PLHA cases cumulatively reported through 2011 and 780,000 PLHA estimated in 2011 [4], suggested that large numbers of PLHA have not yet known their HIV positive status and as a result unknowingly transmit the virus to partners. In the meantime HIV counseling and testing services provided by the CDCs and government hospitals are not well used [12], [13]. Furthermore, 25.4% of those tested and reported HIV positive cases are not in regular follow-up and 45.8% of PLHA are missed in CD4 testing [14]. AIDS mortality remains high and ranked the first among all infectious diseases in China since 2009 [15]. About 30% of PLHA still initiated ART at very late stage despite increased availability of ART services [16].

In the meantime, other social resources, such as civil society organizations and community-based groups, are yet to be closely involved in the response to the challenging HIV/AIDS epidemic. Internationally, task-shifting from health professionals to lay personnel in community-based organizations (CBOs) has been implemented and proven to be effective in provision of HIV counseling and testing, and HIV care and treatment. Studies have shown a lower unit cost, comparable quality and increased acceptability for these services provided by lay personnel, especially peers [17], [18].

Nanjing, the capital city of Jiangsu province, is located in eastern China with a residential population of 6.36 million and a migrant population of 1.75 million [19]. It is also a gay cruising center, where population size of men who have sex with men (MSM) was estimated to be 25,000 in 2008, after the consensus was reached among all relevant stakeholders [20]. This figure was used for all years during the program as there was no any additional estimation effort after 2008. In 2007 the overall HIV/AIDS epidemic of the city was quite low based on HIV/AIDS case surveillance data. As of 2007, the cumulatively reported number of HIV/AIDS cases was about 500, 40.0% due to injection drug use, 22.7% via unprotected heterosexual and 6.5% via unprotected homosexual behaviors. However, a growing HIV epidemic was reported among MSM. HIV prevalence increased from zero in 2003 to 6.6% in 2008, and an HIV incidence of 3.36 per 100 person-years was observed in a cohort study from 2008 to 2010 [21]–[23]. Only one MSM peer group provided hotline-based health education, and conducted outreach intervention services at gay bars and bathhouse during weekends in 2008. HIV testing targeting high risk population, and pre-ART care and treatment referral were mainly implemented by local CDCs, while CBOs and peer groups were not involved.

In 2008, Nanjing started to pilot shifting selected HIV preventive intervention, follow-up care and support tasks along the service chain for HIV programs from health and medical professionals to CBOs, with support from China-Bill & Melinda Gates Foundation Cooperation Project on HIV Prevention and Care. This article aims to describe this ‘task shifting’ and present the findings resulted from it.

## Methods

### Design of the pilot project

After mapping of available CBOs and assessment of their capacity to provide relevant services, two groups of services were selected for ‘task shifting’ from government health facilities, including local CDCs and hospitals, to CBOs. One group was HIV preventive intervention service centered on HIV testing among high risk populations; the other group was follow-up care and support service for PLHA centered on referral for CD4 tests and ART.

A ‘cash on service delivery’ model [24] was adopted for the pilot design. Core components most representative of the two service packages were used as the output measure of achievement for CBOs. Following the performance-based funding mechanism, unit price was developed. For HIV preventive intervention service, per most-at-risk population (MARP) mobilized and screened for HIV and syphilis with the testing result informed, 62 Chinese Yuan (CNY) (approximately 10 USD) were paid to the CBOs. The service package provided by CBOs included health education, condom promotion, HIV and syphilis test mobilization, and pre-and post-test counseling. The core indicator was the number of MARP screened for HIV. Per newly confirmed HIV positive case, an additional of 500 CNY (approximately 82 USD) were paid to the CBOs. The service package provided by CBOs included accompanying and referring patients to local CDCs for HIV repeated test and confirmation, providing social and psychological support while waiting for laboratory results. The core indicator was the number of newly HIV positives confirmed with results informed. For follow-up care and support service for PLHA, 300 CNY (approximately 50 USD) were paid to the CBO who provided a package of services for one HIV positive case for a year, including social support, home-based care, CD4 test facilitation, pre-ART counseling and ART referral. The core indicator was the number of PLHA receiving at least one follow up care, one CD4 test and one syphilis testing within a year. Cash was paid to CBOs every six months by Jiangsu Province Preventive Medicine Association (JSPMA), based on verified core indicators retrieved by respective local CDCs and Nanjing Municipal CDC.

### Implementation of the pilot project

The pilot project started in July 2008 and ended in December 2012 in Nanjing. HIV preventive intervention services covered MSM, female sex workers (FSW) and IDU in 2008 and 2009. After the mid-term project review in 2010, HIV preventive intervention services focused on MSM only, due to evidence of a growing epidemic among MSM and extremely low case detention rates among FSW and IDU. This article focuses on MSM only for the preventive intervention services. As for the follow-up care and support services for PLHA, only local HIV/AIDS cases (Nanjing residents) were included in the pilot program.

To build capacity for the CBOs selected for service delivery, local CDCs and hospitals as well as well-established CBOs were organized and provided training on HIV knowledge, HIV/sexually transmitted diseases, risk behavior change communication, administering rapid HIV test, maintaining confidentiality and procedures for referral and follow-up for PLHA according to national guidelines [25]. For CBOs haven’t been officially registered and without nurses or physicians, rapid HIV testing and counseling were required to implement by peers under the technical support and supervision by local CDCs to avoid adverse events among those who tested HIV positive or social harm. Following pre-and post-test counseling, the window period and need for repeat testing for recent risk and periodic testing every six months with on-going risk were explained, as well as the information of local HIV laboratory algorithm used and the potential discordant testing results between HIV rapid test and confirmatory test, and the need for further repeated testing in six weeks and follow up by local CDCs. Contents of the prevention with positives, pre-ART counseling and ART referral, ART adherence, stigma and discrimination reduction, and program data collection and reporting were also discussed during the training.

### Data collection and verification

Local district CDCs worked with CBOs to collect and enter the programmatic data to the project information reporting system, and compiled and reported the service results to JSPMA every six months. Programmatic data included program management variables such as cost, and performance-based core indicators, such as the number of MARP tested for HIV, the number of newly confirmed HIV positive cases, the number of HIV/AIDS cases received at least one follow up care service within a year and the number of HIV/AIDS patients received at least one CD4 test within a year. These were entered to the web-based national HIV/AIDS information system [26] by local district CDCs. The Nanjing Municipal CDC verified the results of core indicators and shared with JSPMA. JSPMA compared the data from CBOs and Nanjing Municipal CDC, and then paid cash to CBOs based on the verified results.

Non-programmatic data, including the number of total HIV tests and HIV positive cases confirmed and reported, the number of HIV/AIDS cases receiving CD4 tests and initiating ART and being retained on ART at 12-month follow-up, were obtained from Nanjing Municipal CDC through the web-based national HIV/AIDS information system. CD4 testing was provided free of charge once a year for PLHA who were not on ART and twice a year for those who were on ART according to the national guideline, and the criterion for receiving free ART was based on CD4 counts less then 200 cells/uL prior to and in 2011 and then less then 350 cells/uL in 2012 and afterwards [27]. The registered and alive PLHA was used as denominators to calculate the rates of follow-up care and CD4 counts. The PLHA eligible for ART was used as denominators for ART coverage. The cost data for HIV program jointly funded by Chinese central government, Jiangsu Provincial Government and Nanjing Municipal Government were collected annually from Nanjing Health Bureau. The project was approved by the Ethics Committee of the Chinese Center for Disease Control and Prevention.

### Laboratory procedure

HIV rapid test kit (Alere Determine HIV-1/2, Alere Medical Co., Ltd)or enzyme-linked immunosorbent assay (ELISA) kit (Beijing Wantai Biological Pharmacy Enterprise Co., Ltd) were used for HIV screening from finger-prick blood or intravenous blood sample. Blood specimen for screening HIV positive individuals were sent to Nanjing Municipal CDC for confirmation using Western Blot test (HIVBLOT 2.2, Genelabs, Singapore). Following the same testing procedure, CD4 counts were measured using fluorescent activated cell sorter (FACS) analysis with BD FACS Calibur at Jiangsu Provincial CDC in 2008 and 2009, and at the Nanjing Second Hospital, the designated ART hospital in Nanjing since 2010.

### Data analysis

Data is presented annually, prior to and after the pilot launch. Data is disaggregated by and compared among service providers over time. Chi-square was used for trend analysis of categorical data while non-parametric analysis was used for the comparison in median CD4 counts across years. Analyses were performed through SPSS 16.0 version. Statistical significance was defined by P<0.05.

## Results

### Roles of government health facilities and CBOs before and after ‘task shifting’

The scope of the two tasks shifted from government health facilities to CBOs is presented in Table 1. In HIV preventive intervention service, CBOs assumed the functions of three new tasks related to providing rapid HIV testing and result notification, enhancing the roles in two existing tasks of conducting risk behavior change and communication, while government health facilities shifted four tasks substantially to CBOs. In follow-up care and support service for PLHA, CBOs took responsibility for four new tasks, enhanced the roles in two existing tasks of providing social and family support, while government health facilities shifted partially four tasks to CBOs. The roles of stigma and discrimination reduction were both strengthened in government health facilities and CBOs.

**Table 1:** Roles of community-based organizations (CBOs) and government health facilities (GHFs) in the tasks before and after the pilot program in Nanjing, China.

Task	Activities	Role played*			
		Before		After	
		GHFs†	CBOs	GHFs	CBOs
Preventive interventions	Knowledge dissemination	+++	+++	+	+++
	Risk reduction counseling	+++	+	+	+++
	Condom use promotion	+++	+	+	+++
	Referral for testing	+++	−	+	+++
	Rapid HIV testing	+++	−	++	+++
	Notification of test results	+++	−	++	+++
Follow-up care	Social/psychological support	+	+	+	+++
	Home-based care	++	+	++	+++
	CD4 test facilitation	+++	−	++	+++
	Pre-ART counseling	+++	−	++	++
	OI and ART referral	+++	−	++	++
	Drug adherence education	+++	−	++	+++
	Stigma and discrimination reduction	+	+	++	+++

*Role played: Major +++, Medium ++, Minor +, No role–;

† including hospitals and local Center for Disease Control and Prevention.

### HIV preventive intervention services among MSM

After ‘tasks shifting’, the number of CBOs providing preventive intervention services among MSM increased from 1 in 2007 to 8 in 2008 and 10 in 2009 and was maintained at 8 since 2010. HIV testing coverage increased from 4.1% in 2008 to 22.7% in 2012 (P-values <0·001), based on the estimated MSM population size of 25,000 in Nanjing in 2008. In accordance with the increasing HIV testing coverage, the confirmed newly diagnosed HIV cases also increased with the detection rate ranged from 2.6% to 5.8% (P-values <0·001). The baseline CD4 counts of these new cases with Nanjing residence showed a rising trend (P-values <0·01), with the median CD4 cell counts from 309 cells/uL in 2008 to 397 cells/uL in 2012 (Table 2).

**Table 2:** The coverage of HIV testing and HIV detection rate among men who have sex with men, the median CD4 counts among local HIV/AIDS cases, through services by community-based organizations (CBOs), 2008 to 2012,Nanjing, China.

Year	HIV tests performed by CBOs*		Newly detected HIV cases†		Median of CD4 counts‡	
	Number	Coverage %	Number	Rate %	Number of newlydiagnosed local cases	Baseline CD4 counts (cells/µL)
2008	1,034	4.1	27	2.6 (27/1,034)	20	309
2009	2,540	10.2	131	5.2 (131/2,540)	98	356
2010	3,297	13.3	192	5.8 (192/3,297)	149	345
2011	4,547	18.2	158	3.5 (158/4,547)	146	357
2012	5,673	22.7	237	4.2 (237/5,673)	220	397

*P value for trend of the coverage, <0.001; the denominator was 25,000 [20];

† P value for the trend of the rate of newly detected HIV positives, <0.001;

‡ P value for the trend of median CD4 counts, <0.01.

**Figure 1:**
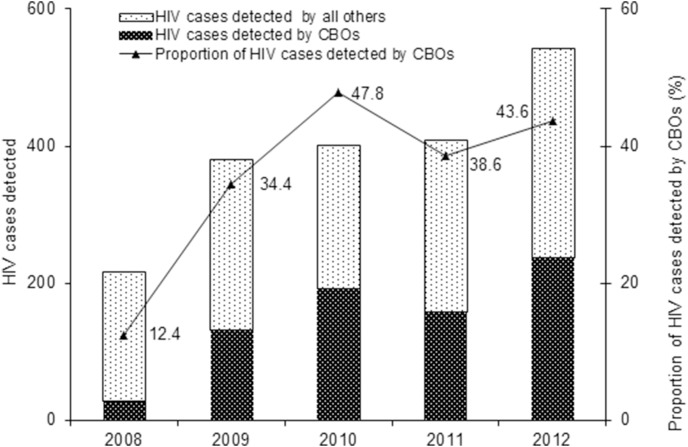
HIV cases detected by community-based organizations (CBOs) and their contribution to the total HIV cases detected, 2008 to 2012, Nanjing, China.

The share of MSM tested for HIV by CBOs out of the total HIV tests performed by government health facilities was very low, accounting for from 0.2% (1,034 of 493,015) in 2008 to 0.7% (5,673 of 799,765) in 2012. However, the total case detected by CBOs each year was very high. The proportion of the cases detected by CBOs out of the total cases detected in the city increased from 12.4% in 2008 to 43.6% in 2012 (Table 3, Figure 1).

**Table 3:** Unit cost per HIV case detected, comparison between government health facilities (GHFs) and community-based organizations (CBOs), 2008 to 2012 (U.S. dollars).

Year	HIV testing		Newly detectedHIV cases		Unit cost per HIV case detected					
					CBOs		GHFs			Unit cost ratio, GHFs/CBOs
	CBOs	GHFs	CBOs	GHFs	Pilot programfunding	Unit cost†	Governmentfunding	HospitalCharges	Unit cost‡	
2008	1,034	493,015	27	190	12,723	471	381,148	2,261,705	13,910	30
2009	2,540	564,965	131	250	36,554	279	340,492	2,718,544	12,236	44
2010	3,297	556,404	192	210	49,248	257	481,475	2,972,977	16,450	64
2011	4,547	712,192	158	251	59,166	374	543,115	3,576,400	16,412	44
2012	5,673	799,765	237	306	77,086	325	616,557	4,098,931	15,410	47
Total	17,091	3,126,341	745	1,207	234,777	315	2,362,787	15,628,557	14,906	47

† All costs by the CBOs to detect one HIV case.

‡ All costs by the government health facilities to detect one HIV case.

The unit cost per HIV case found through CBOs decreased from 2,874 CNY (approximately 417 USD) in 2008 to 1,984 CNY (approximately 325 USD) in 2012. Compared to that through government health facilities, the unit cost per HIV case found through CBOs was 47 times lower (Table 3).

### Follow-up care and support service for PLHA

The number of CBOs providing follow-up care and support service for HIV/AIDS cases living in Nanjing city increased from none in 2007 to 8 in 2012. The percentage of local PLHA receiving follow-up care and support services from CBOs increased from 5.2% in 2008 to 83.5% in 2012 (P-values <0.01). In the meantime, the rate of the CD4 test and ART coverage in Nanjing increased from 71.1% and 78.6% in 2008 to 86.0% and 90.1% respectively (P-values <0.05).The 12-month ART retention rate maintained above 95% since 2009 despite increased volume of ART cohorts (P-values >0.05) (Table 4).

**Table 4:** Local people living with HIV/AIDS (PLHA) covered with follow-up care, tested for CD4, initiated and retained antiretroviral therapy (ART), before and after the task shifting from government health facilities to community-based organizations (CBOs), 2008 to 2012, Nanjing, China.

Year	Enrollment oflocal PLHA alive	CD4 test†		Number of ARTeligibility‡	ART initiation		12 month ARTretention rate§ % (n)
		n	%		n	%	
2008	232	165	71.1(165/232)	84	66	78.6(66/84)	95.5(63/66)
2009	400	287	71.0(287/400)	143	108	75.5(108/143)	96.3(104/108)
2010	572	442	77.3(442/572)	261	188	72.0(188/261)	96.3(181/188)
2011	792	596	75.3(596/792)	408	346	84.8(346/408)	97.1(336/346)
2012	1,090	937	86.0(937/1,090)	574	517	90.1(517/574)	97.7(505/517)

† CD4 test defined as PLHA received CD4 tests at least one time within a year.

‡ The criterion of ART eligibility is CD4 <200 cells/uL prior to and in 2011, and ≤350 cells/uL in 2012 and afterwards.

§ The number of ART initiation was 42 in 2007.

P values for trend analyses: P<0.01 for follow-up care, P<0.05 for CD4 tests, P<0.05 for ART coverage, and P>0.05 for 12-month ART retention rate.

## Discussion

The results along the service chain for HIV prevention and follow-up care program achieved in this pilot, strongly indicate the feasibility of the ‘task shifting’ from government health facilities to CBOs using a ‘cash on service delivery’ approach. Mapping of CBO service providers and assessment of capacity of potential CBO service providers, supplemented with timely and targeted capacity building, appeared to well prepare the CBOs to deliver quality services. There were no any social harm events reported as a result of being tested by CBOs. Procedures established for program management through this pilot appeared to achieve the fundamental objective of ‘cash on service delivery’, ie, performance-based funding. Routine programmatic data generated from CBO services were reported to local CDCs and the program management unit, JSPMA, but actual cash payment made by JSPMA was based on independently verified results through web-based national HIV/AIDS information system, which has been consistently operated since 2005 [26].

The results also indicate the effectiveness of ‘task shifting’ from government health facilities to CBOs, as evident by increased coverage, better targeting and early case detection, as well as increased linkage of detected cases to clinical care, which are highly consistent with findings observed from systematically reviewed community-based tasks recommended by WHO [28]. The coverage of HIV testing among MSM performed by CBOs only increased by 5.5 times and reached 22.7% in 2012, which was almost equal to the country progress reported level of coverage of HIV testing among MSM performed by all health facilities and CBOs combined [29]. HIV detection rate by CBOs remained high regardless of the expansion of the HIV testing during the pilot. The proportion of newly diagnosed HIV cases contributed by CBOs increased remarkably from about one tenth in 2008 to nearly half of the total HIV cases reported in 2012, despite a very low share of HIV tests by CBOs out of the total HIV tests performed each year during the pilot. Both these indicated a good targeting at most at risk subgroups among MSM. With services provided by CBOs and health facilities, in accordance with the national new HIV/AIDS prevention and control approach of ‘Five expands, six strengthens’ [30], coverage of CD4 tests among PLHA increased by about 15%, reached nearly 86% in 2012. The coverage of ART increased from 78.6% in 2008 to 90.1% during the program pilot despite the ART eligibility changed from CD4 <200 cells/µL in 2008 to CD4 ≤350 cells/µL in 2012. Cost-effectiveness was apparent in the pilot, as evident by the 47 times lower unit cost newly diagnosed HIV cases by CBOs compared to that by government health facilities.

In addition, early detection of HIV infection was also found after the greater involvement of CBOs in referral for HIV testing and providing follow-up care services. The median baseline CD4 cell counts increased by about 100 cells/µL in five years and reached nearly 400 cells/µL. This is dramatically different from the current national picture where HIV cases transmitted sexually had a significantly lower median CD4 cell counts than cases infected with other routes of transmission [31]. Early detection of HIV infection reduces not only population level transmission risk, as a result of increased percentage of PLHA knowing their positive status, but provides opportunity for PLHA to receive early treatment to reduce population based viral load in order to achieve more comprehensive HIV prevention [32]–[33].

We recognize the limitations of our data. First, the population size of MSM we used is data estimated in 2008 and hasn’t been updated over time. It may overestimate or underestimate the HIV testing coverage. Second, the data on HIV tests offered by health facilities were not disaggregated by risk groups in the reporting forms, the total number of HIV testing uptake among MSM in the city were not available, thereby CBOs performance on HIV testing and case finding may not be measured accurately. However, the analysis focused on the temporal trend during the pilot. Third, follow-up care and CD4 tests were confined to local resident HIV/AIDS cases in Nanjing, this may not reflect the situation among those temporarily living in Nanjing. These cases may present a different transmission risk portrait from local cases.

Despite limitations, however, this pilot provides evidence that it is feasible to shift tasks from health facilities to CBOs. It is also feasible to adopt ‘cash on service delivery’ model as services resulting from CBOs can be independently verified. CBOs provided more effective services with a lower cost.

While the Chinese government recognizes the significant role CBOs could play in the response to HIV/AIDS epidemic [7], effective mechanism of preparing CBOs and allocating adequate funds to CBOs to provide essential HIV services to high risk groups and PLHA should be implemented. During the past decade, external funders, such as the Global Fund and Gates Foundation, have been the primary sources of support to CBOs to build up capacity and to provide essential services. This pilot provides operational guidance for Chinese government to shift essential HIV services to CBOs in a time when the external donors started to withdraw the development aid from China [34]. The apparent results, that CBOs can provide effective services at a lower cost, shed light on sustainability of providing essential HIV services globally. International aid on HIV/AIDS appears to be leveling out as a result of internal financial constraints in many high-income donor countries [35] while demands of HIV essential services, such as ART, are increasing.
